# Feasibility Study Utilizing NanoString’s Digital Spatial Profiling (DSP) Technology for Characterizing the Immune Microenvironment in Barrett’s Esophagus Formalin-Fixed Paraffin-Embedded Tissues

**DOI:** 10.3390/cancers15245895

**Published:** 2023-12-18

**Authors:** Qurat-ul-Ain, Nicola F. Frei, Amir M. Khoshiwal, Pim Stougie, Robert Odze, Sophie Camilleri-Broet, Lorenzo Ferri, Lucas C. Duits, Jacques Bergman, Matthew D. Stachler

**Affiliations:** 1 Department of Pathology, University of California, San Francisco, CA 94143, USA; quratul.ain@ucsf.edu; 2 Amsterdam UMC Locatie AMC, 1105 AZ Amsterdam, The Netherlands; 3 Department of Pathology, School of Medicine, Tufts University, Boston, MA 02111, USA; 4 Division of Thoracic and Upper Gastrointestinal Surgery, Montreal General Hospital, McGill University, Montreal, QC H3G 1A4, Canada

**Keywords:** Barrett’s esophagus, esophageal adenocarcinoma, tumor microenvironment, spatial transcriptomics, spatial proteomics, immune profiling

## Abstract

**Simple Summary:**

New biological insights into the stepwise development and progression of esophageal adenocarcinoma (EAC) from its pre-cancerous precursor, Barrett’s esophagus (BE), are imperative to develop tailored approaches for the early detection and optimal clinical management of the disease. This study aimed to determine the feasibility of spatially profiling stromal and immunologic properties that accompany the malignant transformation of BE to EAC in standard clinical tissues. NanoString’s GeoMx Digital Spatial Profiling (DSP) technology can detect and quantify protein and ribonucleic acid (RNA) transcripts in a highly multiplexed manner with spatial resolution. Here, we performed a pilot study using DSP, for the measurement of protein and RNA expression on a series of endoscopic samples of BE. We compare a small series of biopsies of non-dysplastic BE (NDBE) from patients who progressed to more advanced disease to patients with NBDE who did not progress, and then perform RNA profiling on samples with a range of histologic diagnoses.

**Abstract:**

Characterization of the Barrett’s esophagus (BE) microenvironment in patients with a known progression status, to determine how it may influence BE progression to esophageal adenocarcinoma (EAC), has been understudied, hindering both the biological understanding of the progression and the development of novel diagnostics and therapies. This study’s aim was to determine if a highly multiplex interrogation of the microenvironment can be performed on endoscopic formalin-fixed, paraffin-embedded (FFPE) samples, utilizing the NanoString GeoMx digital spatial profiling (GeoMx DSP) platform and if it can begin to identify the types of immune cells and pathways that may mediate the progression of BE. We performed a spatial proteomic analysis of 49 proteins expressed in the microenvironment and epithelial cells of FFPE endoscopic biopsies from patients with non-dysplastic BE (NDBE) who later progressed to high-grade dysplasia or EAC *(n =* 7) or from patients who, after at least 5 years follow-up, did not *(n* = 8). We then performed an RNA analysis of 1812 cancer-related transcripts on three endoscopic mucosal resections containing regions of BE, dysplasia, and EAC. Profiling with GeoMx DSP showed reasonable quality metrics and detected expected differences between epithelium and stroma. Several proteins were found to have an increased expression within NDBE biopsies from progressors compared to non-progressors, suggesting further studies are warranted.

## 1. Introduction

Barrett’s esophagus (BE) is the premalignant stage of esophageal adenocarcinoma (EAC) [1,2,3]. EAC remains one of the deadliest forms of gastro-intestinal cancer, with a mortality rate exceeding 90% for advanced disease [4,5]. While the exact process of EAC formation is not fully understood, it is thought to develop sequentially along the metaplasia–dysplasia–adenocarcinoma sequence, with its progression attributed to a series of genomic and epigenetic events that ultimately allow for the clonal selection of BE cells [6,7,8,9,10]. Prior studies on the molecular mechanisms underlying EAC and its stepwise progression from BE have primarily focused on tumor cell-intrinsic features, such as genomic alterations. These studies have made significant progress in our understanding of BE progression [11,12]. However, other extrinsic factors likely also play a role in the progression process. Together with genomic alterations, there is accumulating evidence that the inflammatory microenvironment (iME) plays a role in the progression process by contributing to inhibiting apoptosis, enabling immune evasion, and promoting proliferation, angiogenesis, invasion, and metastasis [13,14]. Studies in the microenvironment of BE have revealed the involvement of lymphoid cells (i.e., T cells, B cells, and NK cells) and myeloid-derived cells (i.e., macrophages, neutrophils, eosinophils), as well as their secreted cytokines and chemokines [15,16]. So far, however, the immune contexture (type, density, and location, as well as phenotypic and functional profile of immune cells) has not been extensively investigated to understand how it may be involved in the progression of BE. A deeper understanding of how BE/EAC–iME interactions contribute to tumorigenesis may direct the development of future diagnostic and therapeutic strategies in high-risk patients to inform clinical decision-making.

While traditional immunohistochemistry (IHC) techniques allow for the spatial profiling of cells, this is often lost when samples are analyzed using broader profiling techniques such as RNA sequencing (either bulk or single cell approaches). To this end, the local cellular proportions, cellular heterogeneity, and deeper spatial distribution are often lacking in tumor microenvironment characterization studies [17]. Combining spatial, cellular composition, and cell state information can aid in identifying micro-niches within the iME that may allow for deeper insights or associations to be identified that can be lost in bulk approaches, such as rarer immune cells within the epithelium versus within the stroma. The determination of the immune makeup within the iME lays the foundation for addressing how the immunological composition and status (activated/suppressed) may dictate BE progression or response to therapy. Therefore, to address this need, proper tissue sampling, along with technologies that retain spatial information while providing hi-plex capabilities, are both required.

In this study, we utilized the GeoMx digital spatial profiler (GeoMx DSP) technology to evaluate its effectiveness in investigating the iME within BE and during progression. The GeoMx DSP is a system for proteomics and transcriptomics analyses in a spatially resolved manner, developed by NanoString Technologies (Seattle, WA, USA) [18]. The GeoMx DSP applies several advanced technologies, including a high-throughput readout, a programmable digital micromirror device for precise area selection, and microfluidic sampling, creating an innovative tool for discovery, translational research, and clinical uses [19].

Our goal was to conduct a pilot study to quantify the abundance of tumor and immune-related transcriptional and protein markers in separate epithelial and stromal compartments, predominately focused on non-dysplastic Barrett’s esophagus (NDBE), but also including samples with dysplasia and early EAC. We utilized the Cancer Transcriptome Atlas (CTA) for high-plex analysis of messenger ribonucleic acids (mRNAs) and a panel of 49 antibodies for protein analysis. We apply this method to study formalin-fixed paraffin-embedded (FFPE) biospecimens selected from a large cohort of NDBE endoscopic biopsies, as well as endoscopic mucosal resections. Furthermore, we aimed to demonstrate the ability of this tool for gastrointestinal (GI) research through investigating the molecular underpinnings and immunomodulatory pathways that underlie BE progression to EAC.

## 2. Materials and Methods

### 2.1. Samples Selection

FFPE blocks of varying ages (7–17 years old) from endoscopic biopsies were selected from a previously described cohort [20] and FFPE blocks of endoscopic mucosal resections (EMR) were retrieved from the pathology archives of the University of San Francisco or the McGill University Hospital system. Fifteen biopsy blocks with a histologic diagnosis of NDBE from seven patients who, after at least two years, progressed to high-grade dysplasia (HGD) or EAC, and eight patients who, after a minimum of 5 years of follow-up, did not progress, were utilized for protein analysis. Three EMR blocks with a range of histologic diagnoses from NDBE to early EAC were utilized for mRNA analysis. The study was performed after IRB approval (UCSF# 19-27460).

### 2.2. Tissue Processing and Hematoxylin and Eosin (H and E) Staining

Five-micron sections were taken from each block. The first and last section was stained with hematoxylin and eosin (H and E) following the procedure previously described [21,22] and was reviewed by trained GI pathologists (MDS) to confirm the histologic diagnosis in each stained section. Supplementary Figure S1 shows representative hematoxylin and eosin images of the endoscopic biopsies (Supplementary Figure S1A) and EMRs (Supplementary Figure S1B).

### 2.3. Sample Preparation and GeoMx Digital Spatial Profiling

The study was performed as part of the NanoString Technology Access Program (TAP) and all slide processing was performed within the NanoString facility (Seattle, WA, USA). Unstained 5 µm thickness tissue sections were shipped to NanoString where they were processed according to previously published protocols for protein and RNA analysis [23].

#### 2.3.1. Spatial Transcriptomics Analysis

To analyze mRNA transcripts in a spatial context, we utilized the human Cancer Transcriptome Atlas (CTA), which profiles 1812 transcripts selected for their relevance in immunology and cancer. Briefly, slides were baked for 2 h at 65 °C for paraffin removal and subsequently loaded onto a Leica BOND RX (Leica Biosystems, Wetzlar, Germany) for tissue rehydration in EtOH and ddH_2_O, heat-induced epitope retrieval (ER2 for 20 min at 100 °C), and proteinase K treatment (1.0 μg/mL for 15 min at 37 °C). The tissue sections were then hybridized with the CTA probes overnight at 37 °C. Following 2× 5 min stringent washes (1:1 4× SSC buffer and formamide), the slides were blocked and then incubated with morphology marker antibodies to guide the region of interest (ROI) selection: PanCK (488 channel, NBP2-33200AF488, Novus Biologicals, Englewood, CO, USA), CD68 (594 channel, sc-20060AF594, Santa Cruz, Dallas, TX, USA), and CD45 (647 channel, NBP2-34528AF647, Novus Biologicals). Syto83 (532 channel, S11364, Invitrogen, Waltham, MA, USA) was used as a nuclear stain. Once the staining was completed, the slide was loaded on to the GeoMx DSP instrument where they were scanned to produce an individual digital fluorescent image based on the morphology markers described above (Supplementary Figure S1C,D), as described in Nanostring protocol [23] to enable morphology marker-guided ROI selection. After ROI selection by a trained GI pathologist (M.D.S.), the ROIs were further sub-segmented into areas of interest (AOI) based on PanCK staining (epithelium vs. stroma), then ultraviolet light was directed by the GeoMx DSP at each AOI, releasing the RNA identification-containing oligonucleotide tags from the CTA probes for collection into a unique well for each AOI. For library preparation, Illumina i5 and i7 dual indexing primers were added to the oligonucleotide tags through PCR to uniquely index each AOI. AMPure XP beads (Beckman Coulter, Brea, CA, USA) were used for PCR purification. The library concentration was measured using a Qubit fluorometer (Thermo Fisher Scientific, Waltham, MA, USA) and the quality was assessed using a Bioanalyzer (Agilent, Santa Clara, CA, USA). Sequencing was performed on an Illumina NextSeq 550 and fastq files were processed into gene count data for each AOI using the GeoMx NGS Pipeline.

#### 2.3.2. Spatial Proteomics Analysis

For protein analysis, unstained sections were interrogated with 49 antibodies focused predominately around immune cell types, immune activation, and immune modulation markers (Supplementary Table S1). Slides for protein analysis were treated similarly for paraffin removal and tissue re-hydration, using standard laboratory protocols [24]. However, antigen retrieval was undertaken using citrate buffer, at a high temperature and pressure. After washing in Tris-buffered saline with tween (TBS-T), slides were blocked for 1 h before morphology marker (same as CTA) addition. GeoMx Human IO antibodies (Immune Cell Profiling, IO Drug Targets, Immune Activation Status, Immune Cell Typing, and Pan Tumor modules) were added for an overnight incubation at 4 °C. After another TBS-T wash, slides were post-fixed in 4% paraformaldehyde (PFA) for 30 min at room temperature (RT). Following an additional wash, slides were stained with ROI selection markers and underwent a similar process for ROI/AOI selection as described above. Post-AOI selection, the oligo tags were cleaved from the panel antibodies and were hybridized with Probe R/U and Plex-Set Reporter Tags, before being counted on an nCounter MAX/FLEX system. The nCounter data (RCC) files were uploaded to the GeoMx for data analysis.

### 2.4. Strategies for Region of Interest Selection and Segmentation in Corresponding Areas of Interest

Using the morphology markers and the paired H and E slide, six individual regions of interest (ROIs) per slide were selected, to encompass both the BE epithelium and the surrounding stroma/lamina propria, of approximately ~3 BE gland profiles in size. Areas which contained squamous epithelium or gastric type glands were avoided.

Using the Pan-Keratin morphology marker, each ROI was sub-segmented into Pan-Keratin positive (PanCK+) AOIs for epithelium and Pan-Keratin negative (PankCK−) AOIs for stroma/microenvironment characterization, as described by Van, T.M. and Blank, C.U [19], which were each analyzed separately (Supplementary Figure S1E,F). Anti-CD45 and anti-CD68 antibodies were utilized to help select representative areas. Once each ROI was selected and segmented into AOIs, slides were processed as described above.

### 2.5. Data Quality Control of Regions of Interest/Areas of Interest and Data Normalization

After quantification of probes, data from the protein and CTA analysis were uploaded to the NanoString GeoMx for quality control (QC), normalization, and analysis.

#### 2.5.1. Protein Quality Control and Normalization

For the protein data, several different methods of normalization were compared to determine the most suitable. These included three housekeeping (HK) proteins (GAPDH, histone H3 and ribosomal protein S6), three immunoglobulin G (IgG) controls including (Ms IgG1, Ms IgG2a and Rb IgG), the number of nuclei, and the overall surface area. IgG and HK normalization account for both differences in ROI size and cellularity [25,26,27].

#### 2.5.2. Cancer Transcriptome Analysis Quality Control and Normalization

CTA data were first checked for successful sequencing. Second, the five probes for each CTA target were filtered for any outliers before consolidation into a single count value per sample per target. Counts were normalized using the third quartile (Q3) normalization method [17,25,26].

### 2.6. Statistical Analysis

After normalization, all statistical analysis and visualization were performed utilizing the built-in GeoMx analysis software (version 2.1). Normalized log2 counts were used for the statistical analyses. For analyzing the expression correlation, the Pearson correlation coefficient (R coefficient) was reported for paired groups: R > 0 indicates a positive correlation, while R < 0 suggests a negative correlation. The expression difference between the two groups was determined using linear mix modeling, while the one-way analysis of variance test was utilized for comparison across multiple groups. Normalized log2 counts were further zero-centralized for generating the heatmap. The Euclidian method was used to calculate the individual distance, and the average hierarchical clustering method was used to determine the expression clusters.

## 3. Results

### 3.1. Feasibility, Normalization, and Quality Control

**Figure 1:**
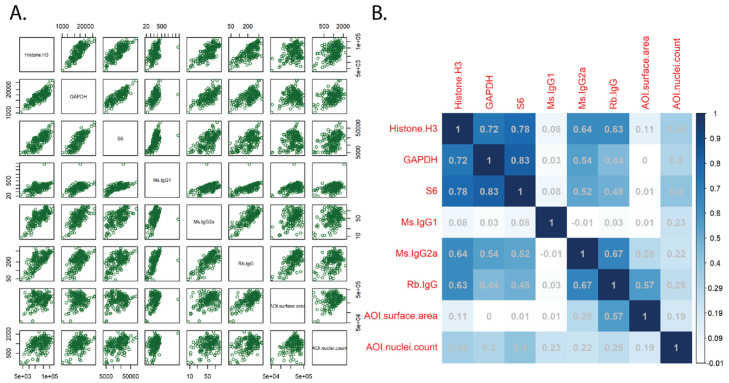
Normalization and QC of protein data. Normalized AOI count plots (**A**) and Pearson correlation coefficients (**B**) of the various normalization strategies, including housekeeping genes (histone H3, GAPDH, ribosomal protein S6), IgG controls (mouse (Ms) and rabbit (Rb)), AOI surface area, and AOI nuclei count. AOI: area of interest.

For protein analysis, multiple methods of normalization were first tested and compared by correlation and standard deviation to determine the optimum approach. The analysis suggested normalization utilizing the housekeeping genes showed a strong correlation and was a good normalization strategy for the protein data (Figure 1); this is what was utilized to analyze the data.

**Figure 2:**
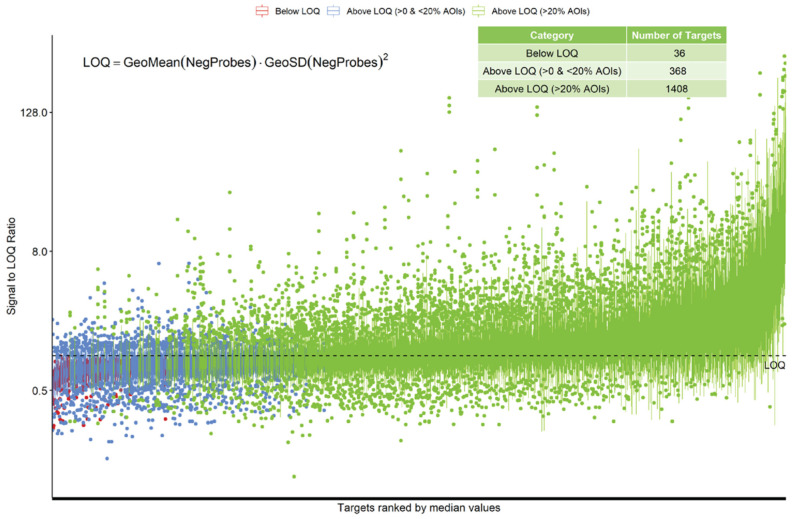
Normalization and QC of RNA data. Individual counts were normalized against the 75th percentile of signal from their own AOI (Q3 normalization) and the limit of quantification (LOQ) was calculated. The normalized signal to LOQ was plotted for each RNA target. Red = below LOQ, blue = above LOQ in less than 20% of AOIs, green = above LOQ in greater than 20% of AOIs. LOQ: limit of quantification.

For RNA analysis, counts from each AOI were analyzed and no sample was considered under-sequenced for further analysis/processing. After normalization, 36/1812 (2%) RNA targets were considered to be below the limit of quantification (LOQ). Either these RNA targets are not expressed in the analyzed samples, or the probes for that target failed. Of the targets, 368/1812 (20%) were detected over the LOQ but in less than 20% of AOIs, potentially showing differential tissue expression or limited successful detection. The rest of the probes, 1408/1812 (78%), were detected above the LOQ in more than 20% of the AOIs (Figure 2).

**Figure 3:**
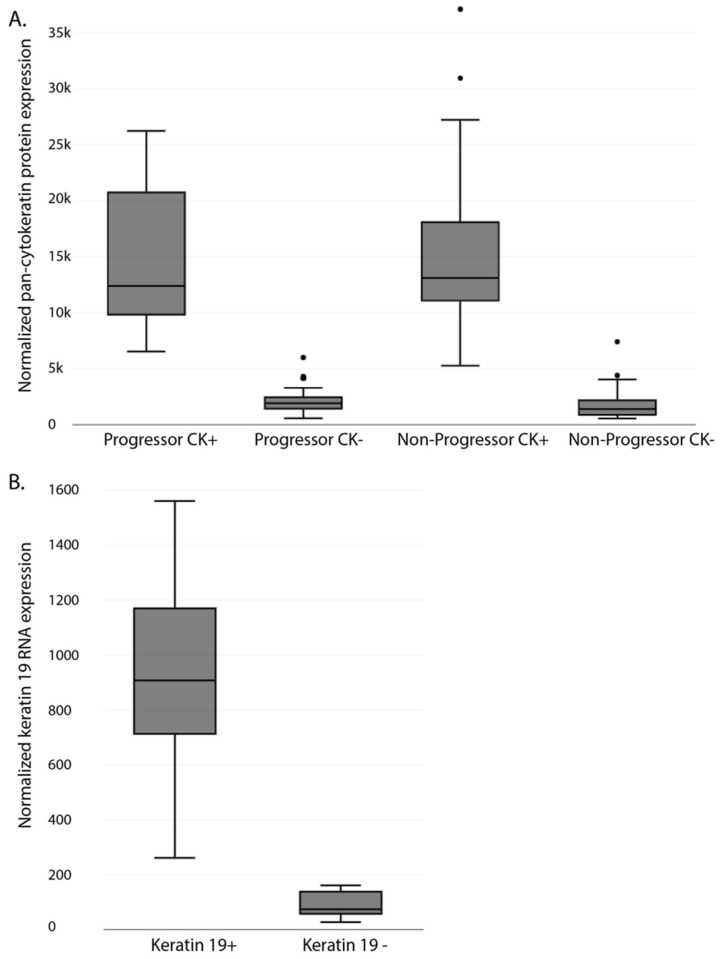
Segmentation of ROIs into Pan-CK+ and Pan-CK− AOIs. Slides were stained with the immunofluorescent morphology marker Pan-CK and used to segment selected ROIs into Pan-CK+ AOIs (Barrett’s epithelium) and Pan-CK- AOIs (surrounding stroma). Box plots show the normalized pan-CK protein expression (**A**) and keratin 19 RNA expression (**B**) of the different AOIs. Protein quantification was separated into progressing and non-progressing patients. CK: Cytokeratin.

To ensure masking and AOI selection separated the epithelium and stromal areas appropriately, the expression of pan-cytokeratin (panCK) for protein and Krt19 for RNA was compared in the two components. As expected, both were highly over-represented in the epithelial compartment (Figure 3A (Protein) and Figure 3B (RNA)).

T-distributed stochastic neighbor embedding (tSNE) plots of the protein data revealed, as expected, a segregation of AOIs by type, with the epithelial AOIs showing in a tight cluster (Supplementary Figure S2A). Additionally, unsupervised clustering of all protein AOIs segregated the epithelial and stromal AOIs (Supplementary Figure S2B). Similarly, for the RNA analysis, when looking at differential gene expression between the Pan-CK+ and Pan-CK− AOIs, the most upregulated genes in the epithelial compartment included keratins and EPCAM, while multiple collagens, immune-related transcripts, and VCAM1/PECAM1 were over-represented in the stromal compartment (Supplementary Figure S3).

### 3.2. Differential Protein Expression in Formalin-Fixed Paraffin-Embedded Samples of Non-Dysplastic Barrett’s Esophagus from Progressing and Non-Progression Patients

**Figure 4:**
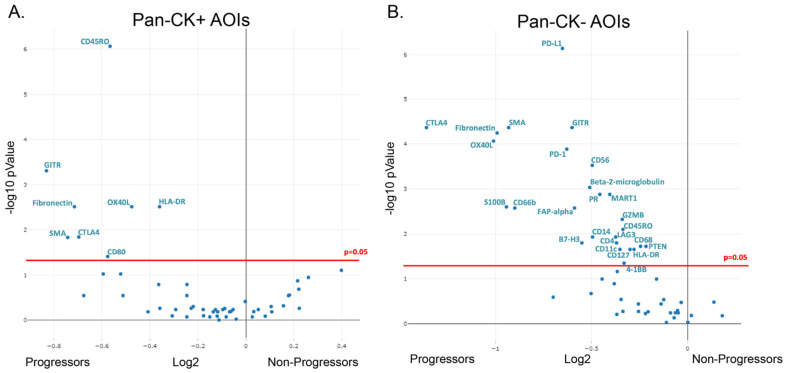
Differential protein expression in progressing and non-progressing BE patients. Volcano plots showing differential protein expression following Benjamini–Hochberg correction for multiple testing in pan-CK+ AOIs (**A**) and pan-CK− AOIs (**B**). CK: Cytokeratin.

For protein analysis, we isolated 42 ROIs from seven patients who progressed and 48 ROIs from eight patients who did not progress. While tSNE and unsupervised clustering was able to separate the epithelial AOIs from the stromal AOIs, it was unable to cleanly separate progressors from non-progressors. However, differential gene expression suggested several differences between progressors and non-progressors, with several proteins being overexpressed in the progression samples (Figure 4). Within the epithelial compartment, several immunoregulatory proteins were upregulated in the progressors compared to the non-progressors, including CD45RO, HLA-DR, and CTLA4, among others. Similar to the epithelial compartment, several immunomodulatory proteins were also found to be over-represented in the stroma of progressors compared to non-progressors, including PD-1, PD-L1, and CTLA4. While not completely specific, in addition to the immunomodulatory proteins, protein markers for several immune cells were also suggested to be over-represented in progressors compared to non-progressors. These included NK cells (CD56, GZMB), granulocytes/neutrophils (CD66b), macrophages (CD68), and CD4 T-cells (CD4) among others. In addition to the immune markers, SMA proteins, associated with myofibroblasts and fibronectin, a protein produced by a variety of cells, including cancer-associated fibroblasts, showed increased expression within the stromal compartment of progressors compared to non-progressors.

### 3.3. Feasibility of Spatial RNA Detection in Formalin-Fixed Paraffin-Embedded Barrett’s Esophagus Samples

**Figure 5:**
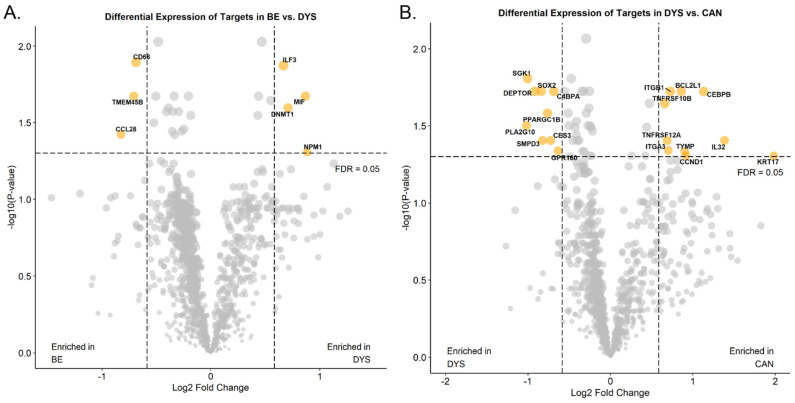
Differential RNA expression across stages of disease. Volcano plots showing differential RNA expression in non-dysplastic BE versus dysplastic samples (**A**) and dysplastic samples versus invasive EAC (**B**). BE: Barrett’s esophagus, DYS: Barrett’s esophagus with dysplasia, CAN: esophageal adenocarcinoma.

To determine how the DSP platform performs when analyzing RNA from endoscopic specimens, four endoscopic mucosal resections with a variety of histologic diagnoses and the CTA were utilized. The CTA interrogates over 1800 RNA transcripts, covering multiple cancer and immune related pathways. Similar to the protein studies, ROIs of approximately similar sizes were selected using immunofluorescence (IF) and a paired hematoxylin and eosin stained slide, and then sub-segmented into AOIs using PanK. A total of 24 ROIs were selected (NDBE *n* = 8, LGD/HGD *n* = 8, and EAC *n* = 8). While the number of samples was extremely small, when comparing the NDBE to the dysplastic epithelial AOIs, several transcripts were upregulated in NDBE compared to dysplastic AOIs, including CD68, TMEM45B, and CCL28, while ILF3, MIF, DNMT1, and NPM1 were upregulated in the dysplastic AOIs (Figure 5A). When comparing the dysplastic to the EAC epithelial AOIs, multiple genes, including CCND1(commonly amplified in EAC), BCL2L1 (apoptosis regulator), and multiple TNF superfamily members, were upregulated in the EAC AOIs (Figure 5B).

## 4. Discussion

Characterization of the tumor microenvironment while maintaining spatial orientation has become an essential resource for the understanding of complex immune cell interactions and the assessment of biomarkers for the prognosis and prediction of immunotherapy response. The NanoString GeoMx DSP platform [17,20,26] allows for the characterization of the tumor microenvironment through the high-plex digital quantification of proteins and mRNA from FFPE tissues with spatial resolution [26]. The application of spatial technologies to large numbers of patient samples that are available in pathology archives as FFPE blocks potentially provides unparalleled insight into cell types, cell location, biomarkers, and the interactions that may underlie disease progression. This technology is rapidly growing and has been applied to cancer samples such as triple-negative breast cancer [27], lung cancer [28], and prostate cancer [29]. However, despite the success of spatial profiling within advanced tumors, pre-cancerous samples, such as NDBE, have been understudied.

To explore the feasibility of studying the NDBE to EAC progression through spatial profiling, we analyzed the expression of 49 proteins and 1800 RNA transcripts within small regions of the epithelium and stroma separately. Using this strategy, segmentation worked well, based on differential expression and clustering analysis. As there is a trade-off between the number of cells profiled and the signal, we benchmarked the performance of the protein panel and the CTA, utilizing areas typically covering ~3 BE glandular profiles (Supplementary Figure S1E,F). While this approach was easy to carry out and provided a relatively large area for analysis, it did sacrifice some resolution.

For protein analysis, we demonstrated that the histone H3, GAPDH, and S6 housekeeping probes correlated across ROIs, and were best used for normalization in protein analysis, while IgG, surface area, and nuclei number were less reliable for normalizing data for quantification. Some previous studies have used area as a normalizer [30,31]. However, area normalization does not take into account cellularity differences like housekeeping genes do. For our smaller biopsies that had variable cellularity, we found surface area and control antibodies to have relatively poor correlation compared to the housekeeping genes. Using the housekeeping genes for normalization, the protein assay seemed to provide results that would be expected and correlated with the transcriptomic analysis. Interestingly, using standard normalization [32] and cutoff metrics for the transcriptome analysis suggested that the RNA profiling worked well, with only 36/1812 (2%) of transcripts considered below the LOQ in FFPE endoscopic mucosal resections. While the tissue pieces within these FFPE blocks are slightly larger than the endoscopic biopsies used for protein analysis, the ROIs that were analyzed were of a similar design and size.

Given the limited number of samples utilized for this pilot, no definitive conclusions can be made. Despite this limitation, our pilot study utilizing NDBE biopsies from patients who would go on to progress to HGD/EAC and patients who did not progress suggested several interesting findings. CD66b (found on neutrophils) and CD68 (found on macrophages) were both increased in NDBE from progressors, suggesting that myeloid cells may be important in the progression process. This finding is in line with a study by Peleg et al., who showed that, a cohort of 13 patients with NDBE or LGD who progressed to a more advanced disease had an elevated neutrophil to lymphocyte ratio, compared to BE patients who did not progress [33]. CTLA4, PD-1, and PD-L1, which have been previously reported to be involved in regulating anti-tumor immunity within several cancers, including EAC [16,34,35], were identified as having increased expression, along with CD45RO and HLA-DR, within NDBE tissues obtained from progressor samples when compared to samples from non-progressors, suggesting that an immunosuppressive environment may develop early in the progression process before invasive cancer develops. Importantly, this also suggests that anti-CTLA4 or anti-PD-L1 immunotherapies might be worth exploring in the setting of early disease, where other mechanisms of immune evasion seen in advanced cancers may not have developed yet. These results require further studies confirming these findings, performing more detailed analysis on the subtypes of cells present, and determining if any differences may be useful as biomarkers for future progression.

## 5. Conclusions

In conclusion, this study highlights the utility of the NanoString GeoMax DSP for profiling relevant proteins and RNAs in small FFPE endoscopic samples. Our pilot study of highly multiplexed measurements of protein expression suggested that several myeloid and immune-modulatory markers were upregulated in biopsies from patients who would go on to progress to a more advanced disease. The application of such novel platforms to provide comprehensive snapshots of clinical samples enables an unprecedented insight into the pre-cancer microenvironment that may be indicative of disease progression.

## Data Availability

Data in this study are found within the manuscript and can be provided by the corresponding author upon reasonable request.

## Supplementary Materials

The following supporting information can be downloaded at: https://www.mdpi.com/article/10.3390/cancers15245895/s1, Figure S1: representative images of Barrett’s esophagus biopsies and EMRs; Figure S2: segregation of AOIs in protein analysis; Figure S3: segregation of AOIs in RNA analysis; Table S1: NanoString TAP Human Immuno-Oncology Protein Panel Content.
