# Supplementary material for: Feasibility Study Utilizing NanoString’s Digital Spatial Profiling (DSP) Technology for Characterizing the Immune Microenvironment in Barrett’s Esophagus Formalin-Fixed Paraffin-Embedded Tissues
Source: Cancers (Basel). 2023 Dec 18;15(24):5895. doi: 10.3390/cancers15245895 (PMC10742302; doi:10.3390/cancers15245895)
Supplement: Supplementary file 1 [file cancers-15-05895-s001.zip › Supp Figure S1.pdf]

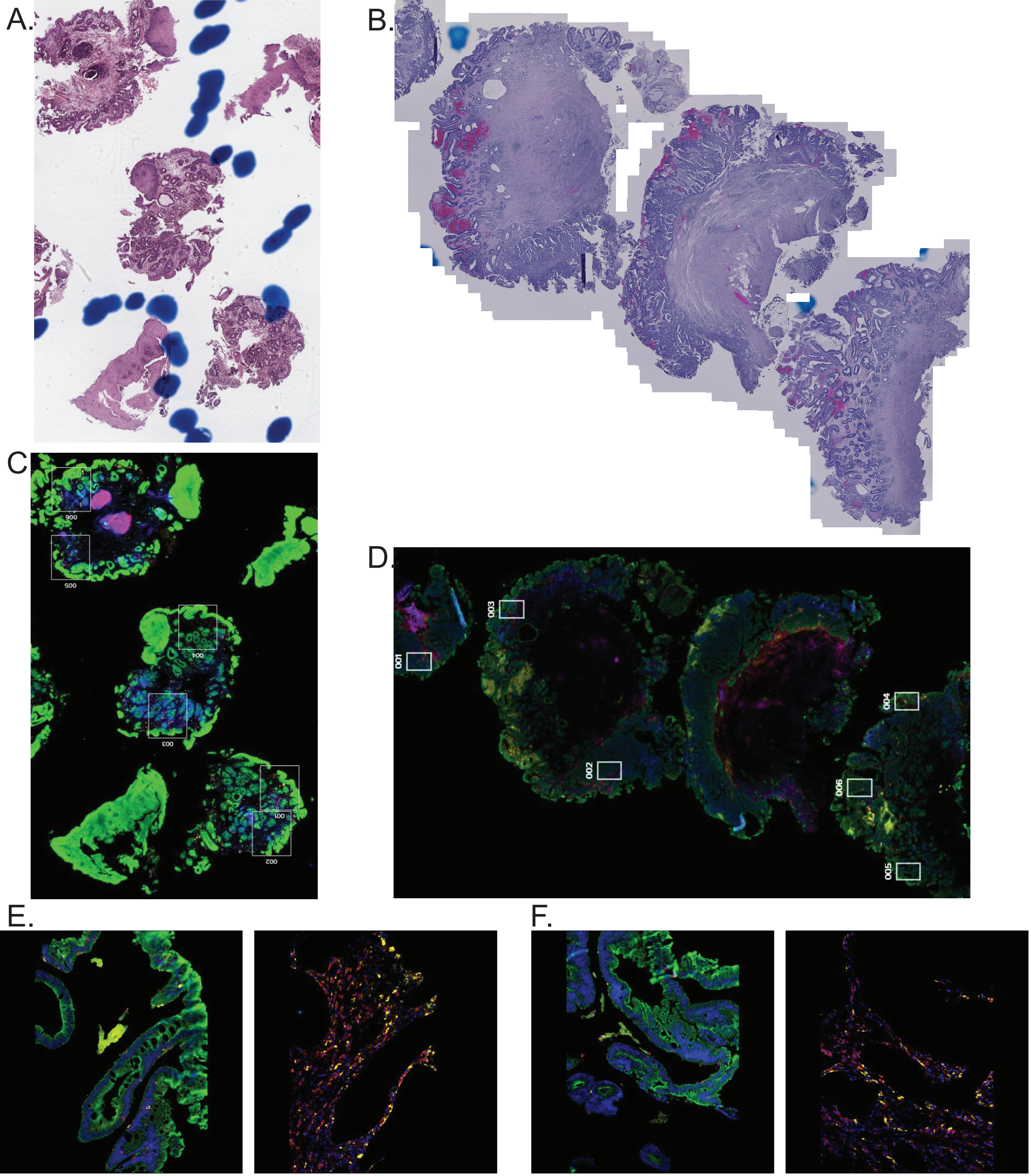

Supplementary Figure S1: Representative images of BE biopsies and EMRs. Hematoxylin and eosin stained sections of endoscopic biopsies (A) and an EMR (B). Immunofluorescent imaging of the morphology markers used to select ROIs (A, biopsy) and (D, EMR). ROI segmentation into Pan-CK positive (left) and Pan-CK negative (right) AOIs in biopsies (E) and EMR (F). For C – F, Green: Pan-CK, Red: CD45, Yellow: CD68, and blue: nuclei.
