# Supplementary material for: Feasibility Study Utilizing NanoString’s Digital Spatial Profiling (DSP) Technology for Characterizing the Immune Microenvironment in Barrett’s Esophagus Formalin-Fixed Paraffin-Embedded Tissues
Source: Cancers (Basel). 2023 Dec 18;15(24):5895. doi: 10.3390/cancers15245895 (PMC10742302; doi:10.3390/cancers15245895)
Supplement: Supplementary file 1 [file cancers-15-05895-s001.zip › Supp Figure S2.pdf]

A.

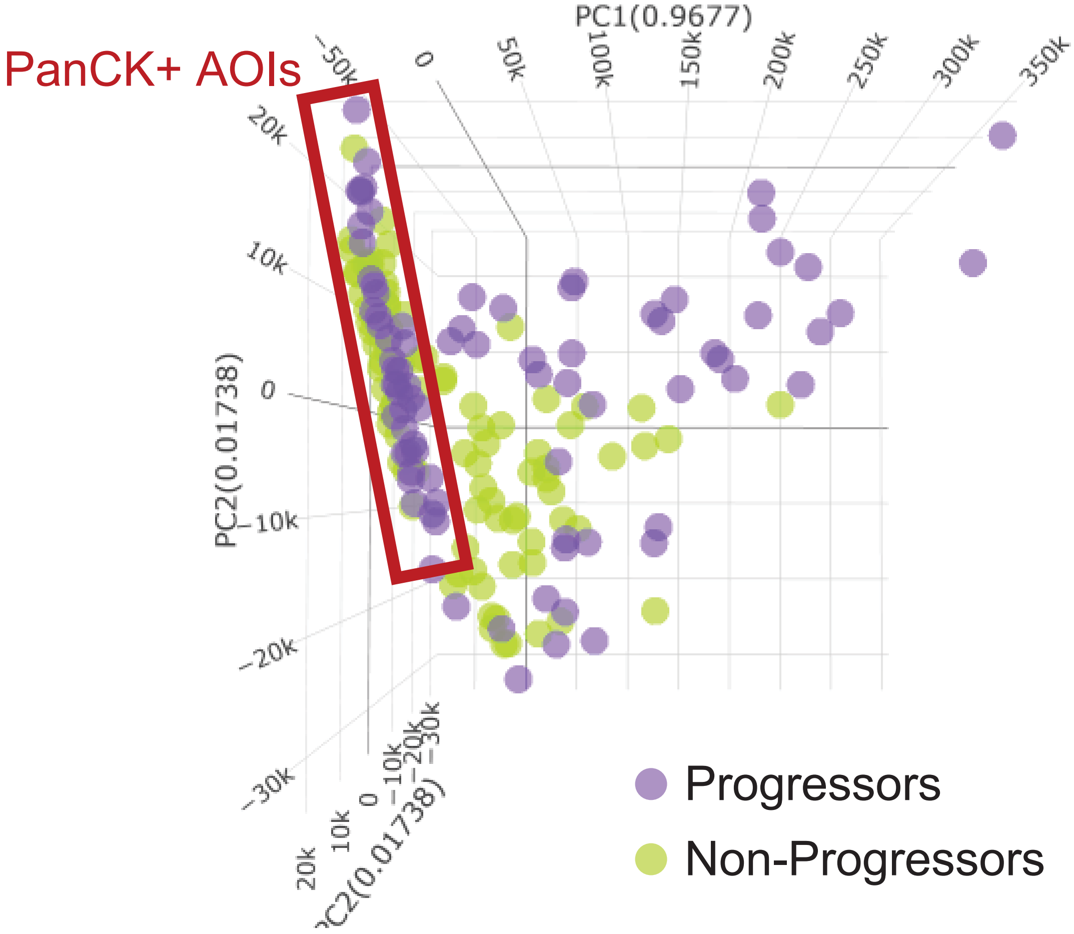

B.

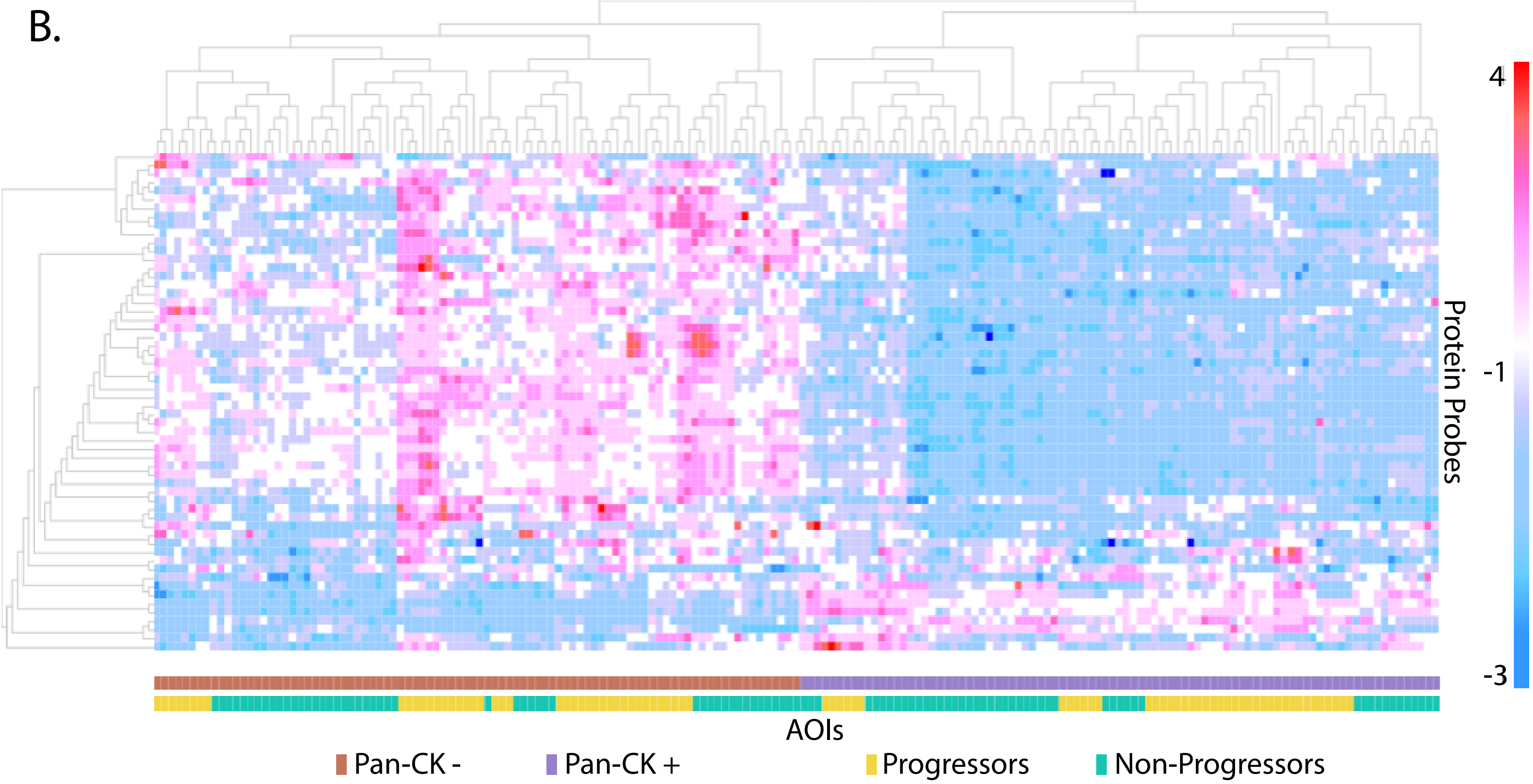

Supplementary Figure S2: Segregation of AOIs in protein a 392 nalysis. (A) Principal component analysis visualized in a three-dimensional tSNE plot. All Pan-CK+ AOIs tightly cluster (red box). AOIs from progressing patients and non-progressing patients do not strongly separate. Purple circles = all ROIs from progressive patients corresponding to group A. Green symbols = all ROIs from group B; non progressors patients. (B) Heatmap view of unsupervised clustering of protein AOIs. Pan-CK status perfectly segregated AOIs whereas progressing patients and non progressing patients had less segregation in both pan-CK+ epithelial and pan-CK- stromal AOIs
