# Supplementary material for: Feasibility Study Utilizing NanoString’s Digital Spatial Profiling (DSP) Technology for Characterizing the Immune Microenvironment in Barrett’s Esophagus Formalin-Fixed Paraffin-Embedded Tissues
Source: Cancers (Basel). 2023 Dec 18;15(24):5895. doi: 10.3390/cancers15245895 (PMC10742302; doi:10.3390/cancers15245895)
Supplement: Supplementary file 1 [file cancers-15-05895-s001.zip › Supp Figure S3.pdf]

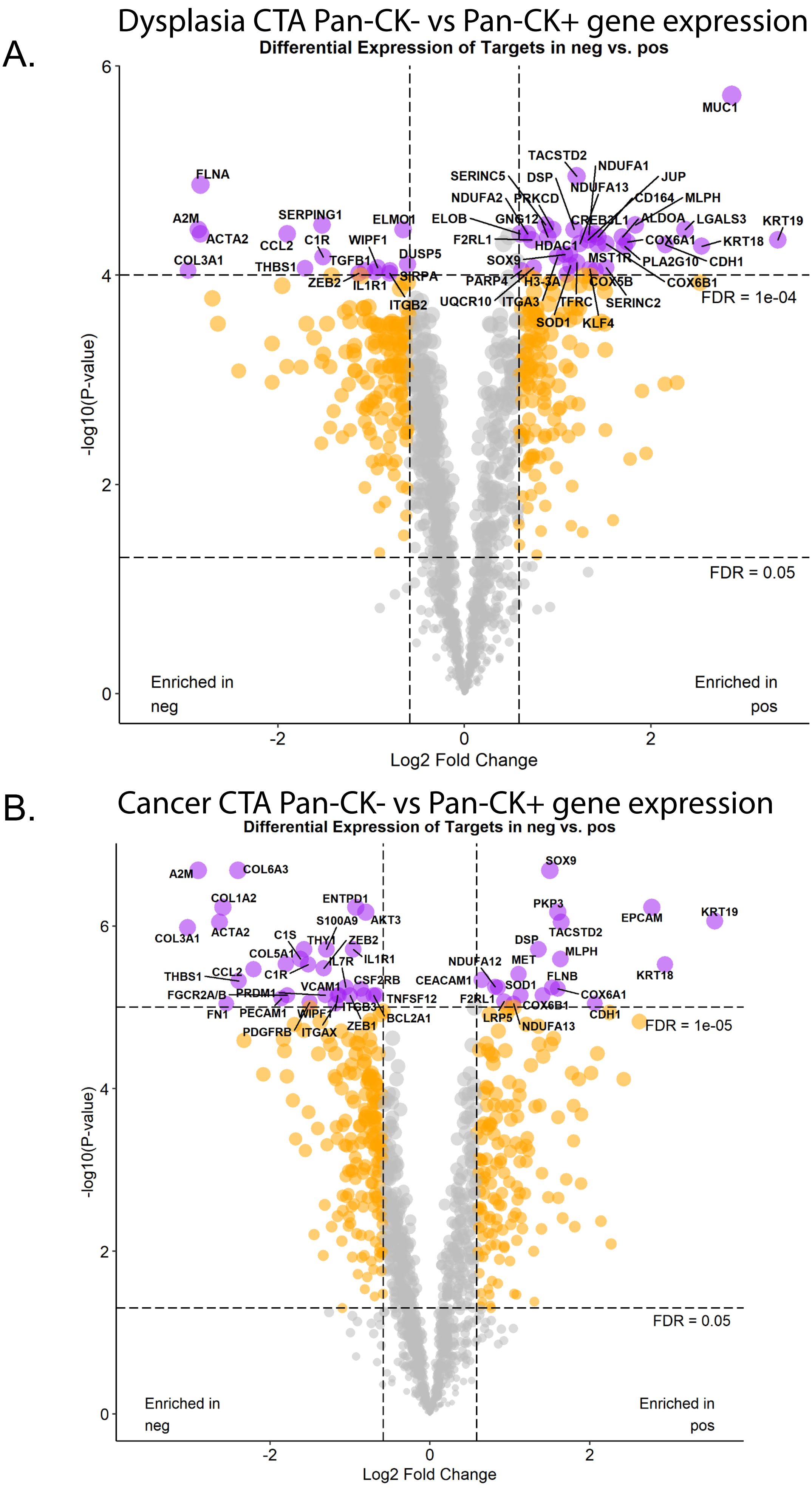

Supplementary Figure S3: Segregation of AOIs in RNA analysis. Volcano plots showing differential gene expression in Pan-CK- versus Pan-CK+ AOIs in dysplasia samples (A) and EAC samples (B).
